# COVID-19 among Healthcare Workers: A Prospective Serological-Epidemiological Cohort Study in a Standard Care Hospital in Rural Germany

**DOI:** 10.3390/ijerph182010999

**Published:** 2021-10-19

**Authors:** Stephanie Hoffmann, Juliane Schiebel, Frank Hufert, Heinz-Detlef Gremmels, Jacob Spallek

**Affiliations:** 1 Department of Public Health, Institute of Health, Brandenburg University of Technology Cottbus-Senftenberg, 01968 Senftenberg, Germany; jacob.spallek@b-tu.de; 2 Institute for Clinical Chemistry, Laboratory Diagnostics and Microbiology, Klinikum Niederlausitz GmbH, 01968 Senftenberg, Germany; juliane.schiebel@klinikum-niederlausitz.de (J.S.); hd.gremmels@klinikum-niederlausitz.de (H.-D.G.); 3 Institute of Microbiology and Virology, Brandenburg Medical School Theodor Fontane, 01968 Senftenberg, Germany; hufert@icloud.com

**Keywords:** SARS-CoV-2, immunity, antibody, healthcare, staff, COVID-19, seroprevalence, pandemic

## Abstract

Healthcare workers (HCW) play a vital role in the SARS-CoV-2 pandemic control. The aim of this study was to assess the prevalence of SARS-CoV-2 antibodies and the risk of COVID-19 infections in a cohort of HCW from four different risk groups (from intensive care unit to administration) of a hospital of a primary care level in rural Germany. The outcomes were monthly measures of antibody seroprevalence over a period of 6 months. Overall, a seroprevalence of 13.41% was determined, with significantly higher prevalence rates among HCW working in areas with more frequent contact to confirmed or suspected cases (30.30%, *p* = 0.003). The group specific differences in the risk of infection from COVID-19 were detected, as HCW groups with frequent exposure seemed to have an increased risk (RR = 3.18, *p* = 0.02; CI95 1.09–9.24). The findings contribute to the epidemiological understanding of the virus spread in an unvaccinated population group, which is highly relevant for the pandemic management.

## 1. Introduction

In 2020, the severe acute respiratory syndrome coronavirus 2 (SARS-CoV-2), the causative agent of the coronavirus disease 2019 (COVID-19), spread internationally. As of 31 December 2020, 1,719,737 people in Germany—41,241 of them in the federal state of Brandenburg—were infected with COVID-19 [1]. While COVID-19 incidences were stabilizing up until late August 2020, the number of newly reported cases steeply increased after October 2020 [1].

Healthcare workers (HCWs) were at specific risk for SARS-CoV-2 because they were caring for increasing numbers of people infected with COVID-19 [2,3]. Gaining evidence about the spread of infection among HCWs is essential to develop targeted protection strategies and to ensure adequate healthcare provision during pandemics. To investigate the number of infections among HCWs, serological testing of specific antibodies against SARS-CoV-2 was commonly used [4,5]. By means of antibody testing, seroepidemiological studies among HCWs in Germany covered different levels of healthcare [6,7,8,9] and different healthcare facilities (e.g., medical supply centers [6], higher care-level hospitals [8], and university hospitals [10,11]). Additionally, studies examined the virus spread among HCWs in healthcare facilities located in sparsely populated rural regions [8] and in urban areas [9].

The primary aim of this serological-epidemiological study, therefore, was to estimate the seroprevalence of antibodies against SARS-CoV-2 among HCWs, in particular at the primary healthcare level in a standard care hospital in a rural German region. The secondary aim was to identify group-related differences by considering different exposure levels defined by working area.

## 2. Materials and Methods

### 2.1. Study Design and Participants

The study was a longitudinal prospective cohort with monthly repeated investigations of (i) blood samples, (ii) exposure history and acute symptoms, as well as (iii) the district-related COVID-19 prevalence and the number of hospitalizations. The study covered a period of six months from July to December 2020, which has been considered as the beginning of the second wave of the COVID-19 pandemic in Germany [12]. The blood samples and data were collected from HCWs in different professions at a standard care hospital in Oberspreewald-Lausitz (OSL), a rural, sparsely populated district in the federal state of Brandenburg in eastern Germany [13].

### 2.2. Laboratory Analysis

The serological testing of total Ig antibodies against SARS-CoV-2 was conducted using a nucleocapsid (N) antigen from a SARS-CoV-2 assay manufactured by Roche (Mannheim, Germany). This technique is based on a double-antigen sandwich principle in which the test sample, the biotinylated SARS-CoV-2-specific recombinant antigen, and the ruthenium-labelled SARS-CoV-2-specific recombinant antigen are incubated together. After the addition of streptavidin-coated microparticles, the complex is bound to a solid phase by the interaction between biotin and streptavidin and transferred to a measuring cell. The microparticles are magnetically fixed, unbound material is removed, and after applying a voltage, the chemiluminescence emission is measured with a photomultiplier. The result is known as a cut-off index (COI), where a COI ≥ 1.00 is interpreted as seroreactive, i.e., positive for SARS-CoV-2 antibodies (99.5% sensitivity, 99.8% specificity).

For HCWs with symptoms, active infection testing (e.g., using polymerase chain reaction) has been integrated into the hospital’s hygiene measures. However, these data could not be linked to the study data due to the lack of possibilities in merging the laboratory identification numbers.

### 2.3. Seroprevalence and Risk Ratio of Antibody Incidence

The antibody seroprevalence at the investigation time points was calculated using the number of seroreactive cases divided by the number of subjects in the four risk groups tested. Additionally, a 6-month seroprevalence among all of the collected blood samples was calculated. Chi-squared tests and Fisher’s exact tests for association were conducted between the dichotomised risk groups (very high risk/high risk, medium risk, lower risk) and the cut-off index (<1.00/≥1.00). Based on two-by-two tables, the point estimates of risk ratios, their confidence intervals (95%), and *p*-values were calculated with reference to the lower risk group.

## 3. Results

In total, 166 participants (85% female) were clustered as follows: emergency room, intensive care unit (very high risk)/cardiology, geriatrics, paediatrics (high risk)/laboratory, radiology (medium risk)/administration (lower risk). The sociodemographic characteristics of the study population (HCWs exclusively) are presented in Table 1. As the systematic vaccination of HCWs in the study hospital started after the end of the data collection (28 December 2020), the participants were neither vaccinated before nor during the study period.

### 3.1. Antibody Seroprevalence

Table 2 represents the SARS-CoV-2 antibody seroprevalence among the participants and the contextual data at the investigation time points. The cohort retention was good. On average, the participants returned for a follow-up five to six times: very high risk group 5.1 (R = 1–6), high risk group 5.6 (R = 1–6), medium risk group 5.5 (R = 4–6), and lower risk group 5.7 (R = 4–6). Thus, the sample sizes varied between the six investigation time points. The seroprevalences were significantly (*p* < 0.05) higher among participants in the very high risk group compared to all other risk groups.

A cumulative 38 of 901 collected blood samples were found to be seroreactive. HCWs in the very high risk group showed a significantly higher 6-month seroprevalence compared to all of the other risk groups (see Table 3).

### 3.2. Risk Ratio of Antibody Incidence

Seroreactive antibodies against SARS-CoV-2 were newly detected at the following investigation time points: in July (*n* = 2), in October (*n* = 1), in November (*n* = 4), and in December (*n* = 15). The risk ratio for antibody incidence was three times higher among HCWs in risk group 4 (*p* < 0.05; 1.09–9.24) (see Table 4). 

## 4. Discussion

From July until December 2020, a broad range of HCWs participated in this follow-up study, who were neither vaccinated before nor during the study period. The aims were to explore the spread of SARS-CoV-2 among this key group in the pandemic management and to estimate the threat of infection in different working areas in a standard care hospital in a rural German region, which is located the federal state of Brandenburg in eastern Germany. As of 4 October 2021, the federal state of Brandenburg reported a percentage of 59% of fully vaccinated individuals [15]. Thus, the findings of this study contribute to still necessary infection prevention measures among HCWs. As the findings are valid for an unvaccinated population group, they also contribute to the epidemiological understanding of the virus spread in HCWs in a pandemic situation and are relevant for the pandemic management in order to reduce (nosocomial) transmissions of SARS-CoV-2 and to ensure sufficient healthcare and health protection (e.g., personal protective equipment) in the current and further pandemics.

During the study follow-ups, the seroprevalence of antibodies increased, leading to an overall seroprevalence of 13.41%. In this study, the seroprevalence was higher compared to serological studies in more urban German districts with sampling at earlier time points in 2020. These studies reported seroprevalences of less than 3.0% among HCWs employed in different healthcare facilities [6,7,11]. This finding reflected the steep increase in confirmed COVID-19 cases in autumn 2020 in the OSL study region, characterized by the number of laboratory confirmed COVID-19 cases (on 15 July 2020: *n* = 53; on 16 December 2020: *n* = 2333) [16]. By this account, the number of people infected with COVID-19 cared for in the study hospital increased (October 2020: *n* = 10; December 2020: *n* = 138) (see Table 2). The seroprevalence of antibodies among HCWs was slightly lower in this study than the seroprevalence of 15.1% among HCWs in a higher care-level hospital in a rural Bavarian district with comparably more hospitalized cases in July 2020 (*n* = 595) [8].

HCWs who frequently reported contact with laboratory confirmed or suspected COVID-19 cases in the course of their work showed a higher burden of infection than the staff in other working areas, which indicates differences between occupational groups, such as administration employees and frontline HCWs within the hospital. This finding is supported by serosurveys of HCWs in hospitals with a large number of inpatient beds in urban regions. Here, seroprevalences were higher among the frontline HCWs [9] or among the staff working on wards without “known or suspected COVID-19 patients” [11] (p. 2). In contrast, Bahrs et al., 2020, did not find any association of antibody seroprevalence and the working area in a sample of a university hospital [10]. The contradictory data on risk differences might be due to different reasons.

First, the comparison of the findings could be challenging due to variations in the used material (i.e., blood samples, respiratory samples [5,17]) and their laboratory analyses (e.g., polymerase chain reaction, antibody tests [5,18]) to determine the infection rates among HCWs. This study used chemiluminescence-based immunoassays for analysing the presence of SARS CoV-2 antibodies (comparable to Bahrs et al., 2020, [10]).

Second, the compliance to the implemented hygiene guidelines might contribute to different infections rates among HCWs. Bahrs et al., 2020, argue that their findings might be due to ample access to and use of personal protective equipment (PPE) [10]. Similarly, a literature review of seroprevalence surveys summarized international findings and concluded that the use of appropriate PPE reduces nosocomial transmissions of SARS-CoV-2 [4]. Korth et al., 2020, suppose the differences in individual awareness of human to human transmission of SARS-CoV-2 contribute to the inefficiency of hygiene standards [11]. The adherence to mandatory mouth and nose coverage or the extent of an inappropriate use of PPE (e.g., reuse [19]), however, was not evaluated in this study.

Serological screening for antibodies reactive to a specific virus (e.g., human novel coronavirus) have been shown to be applicable for contact investigations among HCWs in previous infection control strategies [20]. The sensitivity and specificity of the antibody test used in this study were high and in line with regular antibody tests [5]. A limitation was that the COI is not indicative of the total amount of antibodies in the sample, nor does it evaluate the effectiveness of the antibodies [21,22]. A lower sensitivity within the first week since the onset of symptoms did not allow for an accurate estimation of the virus spread [3]. While the follow-up design buffered such diagnostic issues, the associations with self-rated exposure might have been undetected.

## 5. Conclusions

In a standard care-level hospital located in a rural, severely affected region, the seroprevalence of SARS-CoV-2 antibodies among HCWs corresponded with the dynamic of infection processes within the population and with the number of inpatient treatments. This study indicates a variation in the risk of getting infected with SARS-CoV-2 by working area. Although these findings contribute to the epidemiological understanding of the virus spread in this population group, further research about long-term immunity and about the reasons for group-related differences is needed.

## Figures and Tables

**Table 1:** Sociodemographic characteristics of the study population (*n* = 166).

Risk Group *	Very High Risk (4)		High Risk (3)		Medium Risk (2)		Lower Risk (1)	
	*n*	%	*n*	%	*n*	%	*n*	%
	33	19.88	54	32.53	35	21.08	42	25.30
Female	26	78.80	49	90.70	32	91.40	34	81.00
Age, mean, y	42.82		46.77		45.31		45.31	
20–29 y	3	9.09	5	9.26	2	5.71	2	4.76
30–39 y	12	36.36	9	16.67	11	31.43	9	21.43
40–49 y	10	30.30	13	24.07	5	14.29	16	38.10
50–59 y	5	15.15	20	37.04	13	37.14	14	33.33
60–69 y	3	9.09	9	11.11	4	11.43	1	2.38
Missing	0	0	1	1.85	0	0	0	0
Profession								
Nursing staff	23	69.69	33	61.11	1	2.86	0	0
Physician	4	12.12	8	14.81	0	0	0	0
Therapist	2	6.06	10	18.52	1	2.86	0	0
Lab assistant	0	0	0	0	15	43.86	0	0
Radiology assistant	0	0	0	0	8	22.86	0	0
Diagnostics staff	0	0	0	0	3	8.57	0	0
Patient service staff	0	0	3	5.55	0	0	0	0
Social service staff	0	0	0	0	0	0	3	7.14
Administrative staff	0	0	0	0	2	5.71	36	85.70
Missing	4	12.12	0	0	5	14.29	3	7.14

Note: *n* = Sample size; * risk group information missing *n* = 2.

**Table 2:** SARS-CoV-2 antibody seroprevalence among participants and contextual data at investigation time points.

Risk Group	July		August		September		October		November		December		Overall	
	No./*n*	P	No./*n*	P	No./*n*	P	No./*n*	P	No./*n*	P	No./*n*	P	No./*n*	P
4 Reference	1/30	3.33	1/30	3.33	1/27	3.70	2/26	7.96	3/26	11.54	10/29	34.48	10/33	30.30
3	1/54	1.85	1/50	2.00	1/51	1.96	1/51	1.96	3/48	6.25	8/48	16.67	8/54	14.81
2	0/31	0.00	0/34	0.00	0/33	0.00	0/33	0.00	0/34	0.00	0/29	0.00	0/35	0.00
1	0/41	0.00	0/42	0.00	0/41	0.00	0/38	0.00	1/42	2.38	4/37	10.81	4/42	9.52
Total	2/158	1.27	2/156	1.28	2/152	1.31	3/148	2.03	7/151	4.63	22/143	15.39	22/164	13.41
Fisher’s exact test	*p* = 0.349	-	*p* = 0.349	-	*p* = 0.325	-	*p* = 0.080	-	*p* = 0.101	-	*p* = 0.003	-	*p* = 0.003	-
**Contextual Data on Number of Confirmed Cases of COVID-19**														
	DC/Inh.	P	DC/Inh.	P	DC/Inh.	P	DC/Inh.	P	DC/Inh.	P	DC/Inh.	P	DC/Inh.	P
Cumulative prevalence in district	58/108,396 *	0.05	69/108,396	0.06	72/108,396	0.07	148/108,396	0.14	714/108,396	0.66	2333/108,396	2.15	2333/108,396	2.15
Hospital cases	2	-	0	-	0	-	10	-	73	-	138	-	223	-

Note: DC = Number of cumulative COVID-19 cases in the district; Inh. = Inhabitants living in the study region; *n* = Sample size; No. = Number of participants found to be seroreactive (i.e., cut-off index ≥ 1.00); P = Prevalence; * As of 31 December 2020, 108,396 inhabitants lived in the study region that reflects a population decrease by *n* = 957 in year 2020 [14].

**Table 3:** SARS-CoV-2 antibody 6-month seroprevalence among blood samples.

Risk Group	No./*n*	6-Month Prevalence	Chi-Squared Test
4 Reference	18/166	10.84	χ^2^ = 21.96 *p* = 0.000 φ = 0.16
3	15/300	5.00	
2	0/193	0.00	
1	5/239	2.10	
Total	38/901	4.22	-

Note: *n* = Sample size; No. = Number of blood samples found to be seroreactive (i.e., cut-off index ≥ 1.00).

**Table 4:** Incidence of detectable antibodies among participants, self-rated exposure, and acute symptoms within the last four weeks before detection.

Risk Ratio (RR) of New Cases	Risk Group 4 *n* = 10	Risk Group 3 *n* = 8	Risk Group 2 *n* = 0	Risk Group 1 *n* = 4
RR; (*p* value; 95.00% CI)	3.18 (*p* = 0.02; 1.09–9.24)	1.55 (*p* = 0.44; 0.50–4.81)	-	1.00
Data on New Cases	No./%	No./%	No./%	No./%
Self-rated Exposure				
Case contact on job	8/80.00	6/75.00	-	1/25.00
Case contact off job	1/10.00	2/25.00	-	1/25.00
Suspected contact on job	5/50.00	5/62.50	-	1/25.00
Suspected contact off job	2/20.00	0/0.000	-	0/0.000
Stay in outbreak area	2/20.00	2/25.00	-	0/0.000
Stay abroad	0/0.000	1/12.50	-	0/0.000
Attended an event	0/0.000	2/25.00	-	1/25.00
Self-rated Acute Symptoms				
Asymptomatic	0/0.000	2/25.00	-	1/25.00
Fever	2/20.00	2/25.00	-	1/25.00
Coughing (expectoration)	3/30.00	3/37.50	-	1/25.00
Coughing (dry)	6/60.00	4/50.00	-	2/50.00
Sore throat	6/60.00	6/75.00	-	2/50.00
Fatigue	9/90.00	5/62.50	-	3/75.00
Rhinitis	6/60.00	4/50.00	-	2/50.00
Gastrointestinal distress	4/40.00	2/25.00	-	1/25.00
Odour/taste disorders	6/60.00	3/37.50	-	2/50.00

Note: No. = Number of participants with positive rating (multiple answers possible).

## Data Availability

The datasets generated and analysed during the current study are not publicly available.

## Abbreviations

COVID-19	coronavirus disease 2019
COI	cut-off index
HCW	healthcare worker(s)
OSL	study region Oberspreewald-Lausitz
